# Counselling patients with sudden, irreversible sight loss

**Published:** 2017-03-03

**Authors:** Jasmine Thombs, Louise de Board

**Affiliations:** 1 Senior Nurse Counsellor and Joint Manager: Patient Support Service Team, Moorfields Eye Hospital, London, UK.; 2 Senior Nurse Counsellor and Joint Manager: Patient Support Service Team, Moorfields Eye Hospital, London, UK.

**Sudden loss of vision is devastating to the patient and close relatives. This article discusses how to talk with someone who has lost their vision and how to help them with their concerns and questions.**

**Figure:**
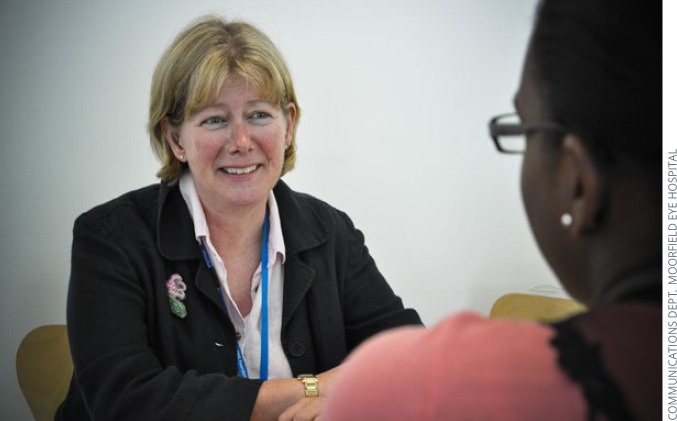
Counselling a patient. UNITED KINGDOM

Sudden loss of sight is a life-changing event that changes the patient's view of the world and how they perceive themselves and others. In this article we will consider the emotional and psychological Impact of those affected by sudden and Irreversible sight loss.

## Grief and loss

It is recognised that people affected by sight loss will understandably experience very strong and at times over whelming feelings of grief and loss. In cases of sudden loss the reaction is often severe. Patients often experience panic and confusion, making it difficult to assimilate information and to make rational decisions. For the majority of patients with diagnosed sight-threatening conditions, the reality is feelings of helplessness, hopelessness and fear. These are often compounded by insomnia, loss of appetite, palpitations, visual hallucinations, aggression, anger, tension, frustration, disorganisation, irritability, restlessness, Inability to concentrate, apathy, and depression.

## Case study ‘Linda’ a patient, affected by sudden irreversible sight loss

Linda is fifty years old, she is single and previously worked as a designer. She has had glaucoma for many years. Nine months ago her vision started to deteriorate. Following a period of further investigation and treatment Linda was assessed as having near complete sight loss.

Linda spent her first counselling sessions talking about her losses and frustrations since her sudden sight loss. Linda says that she is going out less often and admits to feeling lonely and isolated; she is feeling scared and fearful about the future. Her mood is low and at times she feels very depressed. Linda says that her loss of sight doesn't feel real and she keeps hoping that it will improve. Linda is angry about the impact her condition is having on her independence. Linda says she is particularly worried about becoming a burden to others.

It is common for patients with sudden sight loss to feel blame in relation to anger. Sometimes patients feel that more should have been done. In Linda's case at first she initially blamed herself, for not noticing her symptoms earlier.

**“You are just a visitor passing through my country”**, a statement made by a patient who had experienced sudden sight loss. It illustrates how patients can feel that at times, healthcare professionals do not understand the psychological impact of sudden sight loss.

### Rehabilitation, stigma and shame

Linda was reluctant to use any aids to help with her sight loss including a white stick or cane. She was reluctant to work with the staff to begin her rehabilitation. She spoke of feelings of stigma and shame.

After some months Linda agreed to register as severely sight impaired. Registering with a certificate of vision impairment enables people in the UK to receive certain benefits from social services and tax concessions. But the acknowledgement of being registered as blind (severely sight impaired), often has a huge emotional impact, as it confirms that the sight loss is permanent. Eventually Linda decided that she was going to start rehabilitation training in the form of long cane training. She also attended a course entitled learning to live with sight loss' at the National Association for the Blind.

Like all people affected by sudden sight loss, Linda Is going through an on-going period of adjustment. She admitted that her certificate of vision impairment registration was significant in the ongoing process of her adjustment to her sight loss

### Summary

Adjusting to sight loss is hard and exhausting work, and is an active process. The psychological impact can be frightening and disturbing but it is the process by which a person can heal.

Linda said that she found the counselling sessions useful as they gave her space to think and to talk things through. In the sessions, she had been able to talk freely, express her anger, cry and vent her feelings in a way that she had not able to do with her friends or family.

As counsellors, we recognise that people who experience sudden sight loss have to be ready emotionally before they can begin rehabilitation. We talk of patients affected by sight loss going through an on-going period of adjustment involving re-conceptualisation of self, perhaps ultimately leading to acceptance.

